# De Novo Transcriptome of *Mammillaria bombycina* (Cactaceae) under In Vitro Conditions and Identification of Glyoxalase Genes

**DOI:** 10.3390/plants11030399

**Published:** 2022-01-31

**Authors:** Carolina Enríquez-González, Cristina Garcidueñas-Piña, Osvaldo Adrián Castellanos-Hernández, Sergio Enríquez-Aranda, Abraham Loera-Muro, Gilberto Ocampo, Eugenio Pérez-Molphe Balch, José Francisco Morales-Domínguez

**Affiliations:** 1 Centro de Ciencias Básicas, Departamento de Química, Universidad Autónoma de Aguascalientes, Av. Universidad 940, Aguascalientes 20100, Mexico; al687@edu.uaa.mx (C.E.-G.); cristina.garciduenas@edu.uaa.mx (C.G.-P.); eperezmb@gmail.com (E.P.-M.B.); 2 Centro de Investigación en Biología Molecular Vegetal, Centro Universitario de la Ciénega, Universidad de Guadalajara, Av. Universidad, 1115, Linda Vista, Ocotlán 47810, Mexico; ocnoscr@gmail.com; 3 Centro de Ciencias Básicas, Departamento de Sistemas de la Información, Universidad Autónoma de Aguascalientes, Av. Universidad 940, Aguascalientes 20100, Mexico; sergio.enriquez@edu.uaa.mx; 4 CONACyT Centro de Investigaciones Biológicas del Noroeste S.C., Av. Instituto Politécnico Nacional 195, Playa Palo de Santa Rita Sur, La Paz 23096, Mexico; aloera@cibnor.mx; 5 Centro de Ciencias Básicas, Departamento de Biología, Universidad Autónoma de Aguascalientes, Av. Universidad 940, Aguascalientes 20100, Mexico; gilberto.ocampo@edu.uaa.mx

**Keywords:** Cactaceae, Glioxalases, D-lactate, methylglyoxal, RNA-Seq

## Abstract

*Mammillaria bombycina* is a cactus distributed in the central region of Mexico. Cactaceae have the particularity of surviving drought and high temperatures, which is why in vitro propagation studies have been carried out successfully to preserve this species and use it as a study model in cacti. In this contribution, a de novo transcriptome of *M. bombycina* was produced under in vitro conditions for the identification and expression of genes related to abiotic stress. Samples were sequenced using an Illumina platform, averaging 24 million clean readings. From assembly and annotation, 84,975 transcripts were generated, 55% of which were unigenes. Among these, the presence of 13 isoforms of genes belonging to glyoxalase I, II and III were identified. An analysis of the qRT-PCR expression of these genes was performed under in vitro and ex vitro conditions and dehydration at 6 and 24 h. The highest expression was observed under greenhouse conditions and dehydration at 24 h, according to the control. The de novo assembly of the *M. bombycina* transcriptome remains a study model for future work in cacti.

## 1. Introduction

**Figure 1:**
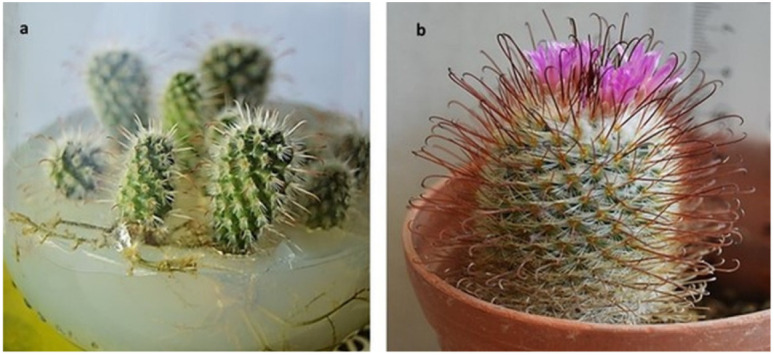
*Mammillaria bombycina*. (**a**) specimens grown in vitro for 18 months and (**b**) ex vitro in a greenhouse.

*Mammillaria bombycina* is located in the mountainous area between the states of Jalisco and Aguascalientes, Mexico in the subtropical scrub, and in the low deciduous forest of this region [1]. It is generally small, globose, with abundant thorns and very colorful flowers (Figure 1), which makes it highly attractive [2]. Overexploitation, overgrazing, and climate change have considerably reduced its spread and natural distribution [3], which is why *M. bombycina* has been classified as endangered by Mexican authorities in NOM-059. -SEMARNAT-2010 [4]. As measures to preserve this species, a large number of in vitro propagation protocols have been developed (Figure 1a) as well as a germplasm bank [5]. *M. bombycina* seedlings grown in vitro can adapt to an ex vitro medium, and their cultures can be maintained under greenhouse conditions (Figure 1b), enabling studies of the identification and expression of genes related to abiotic stress to be developed [6] and research related to its metabolism to continue.

*M. bombycina* molecular analyses can provide information on the mechanisms of adaptation of the species to extreme conditions. In other plant species, proteins, transcription factors, osmoprotectors, and secondary metabolites have been identified as important components in abiotic stress response [7,8]. Under these conditions, the overproduction of glyoxalases has been detected [9]; these enzymes are responsible for metabolizing methylglyoxal (MG) to D-lactate [10]. MG is a component that is synthesized from metabolic intermediates of photosynthesis, glycolysis, protein metabolism, and lipid peroxidation [11]. However, when plants are under some types of stress, large amounts of MG form, which is toxic to cells due to its ability to act as a glycation agent and increase reactive oxygen species (ROS) [9,12,13]. There are two systems of glyoxlases: glyoxalase I (GLYI; lactoylglutathione lyase) and glyoxalase II (GLYII; hydroxyacylglutathione hydrolase) that, together, catalyze the isomerization of MG to D-lactate, using reduced glutathione (GSH) as a cofactor. On the other hand, glyoxalase III (GLYIII) converts MG to D-lactate in a single step, without the need to reduce glutathione or requiring the intervention of a cofactor or metal ions for its activity [14].

The application of next-generation sequencing (NGS) technology has brought substantial advances in the genomic research of many species. This technology has also been applied for more than a decade to the analysis of transcriptomes for the discovery and analysis of genes [15]. This ability has made it possible to determine and understand the expression patterns of genes in response to different stress conditions. When information on reference genomes or transcriptomes is lacking in the expression analysis of non-model plants, it is highly important to analyze the de novo transcriptome because, by means of bioinformatics tools, it is possible to reach the assembly of short sequences and obtain complete information on the expressed genes [16,17]. Several de novo transcriptomes have been reported from non-model organisms, particularly from succulent plants; for example, in *Lophophora williamsii* for the search for genes involved in the synthesis of mescaline [18], in *Agave sisalana* for the study of genes under drought stress [16], in *Agave* H11648 in the identification of cellulose synthase genes [19], in *Hylocereus undatus* for the analysis of floral induction [20], in *Agave deserti* and *Agave tequilana* in the search for genes related to drought tolerance [21], and in *Pachycereus pringlei* for the regulation of root apical meristems [22].

In this study, we report the de novo transcriptome and identification of genes expressed under in vitro conditions in *M. bombycina.* Using the transcriptome assembly, genes were compared with different de novo transcriptomes. In addition, genes coding for glyoxalases were identified. A detailed bioinformatics study was conducted as well as an analysis of expression in vitro, ex vitro, and in dehydration conditions at 6 and 24 h. This is the first transcriptome under in vitro conditions of *M. bombycina* to be studied and could, therefore, expand our knowledge to identify and analyze an extensive number of genes in this or other species of cacti under different conditions.

## 2. Results

### 2.1. Illumina Sequencing, Trimming, and Filtering of the Readings Obtained

From the total RNA of the aerial part of *M. bombycina* plants in vitro, the libraries were constructed for each of the three biological repeats for their sequencing by Ilumina. From these libraries, approximately 24 million readings were obtained (Table S2). The quality and integrity of the raw reads of the sequences were determined using the FastQC tool and showed a quality score >35 Phred (average score in all sequences). Trimming and filtering of the readings were performed with the Fastp program, with which adapter remnants were eliminated, and automatic PloyG trimming was carried out (Table S3). The information discarded from each of the sequences was highly accurate because approximately 90% of the readings were retained and used in the assembly (Table S4).

### 2.2. Assembling the De Novo Transcriptome with the Trinity Program

Filtered reads were concatenated on paired endpoints (in single files ‘left.fq’ and ‘right.fq’, respectively) so that they could be digitally standardized and assembled de novo. This approach resulted in a total of 79,881 Trinity-generated transcripts, with a total of 78,412,335 assembled bases. Of the total transcripts, 47,406 correspond to unique genes. Based on the lengths of the assembled contigs of the transcriptome, the standard Nx length statistic was calculated. In this way, it was determined that 50% of the nucleotides of the assembly are found in contigs that are at least 1574 bases long, with an average of 981 bases per contig. As for the longest or most representative isoforms per gene, there are a total of 37,675,612 assembled bases, of which 50% of the nucleotides are in contig that are at least 1417 bases long, with an average of 794 bases (Table 1).

### 2.3. Annotation of M. bombycina Transcriptome

Table 2 describes the results obtained from the functional annotation of the de novo transcriptome of *M. bombycina*, where the statistical summary generated from the Trinotate.xls database is observed. A total of 84,975 transcripts were obtained, 55% of which are unique (47,406 unigenes). In the results obtained for Blastx, a total of 43,640 hits of nucleotide sequences similar to others already annotated in the aforementioned databases were generated, of which 38,175 are unigenes. In the case of Blastp, a total of 34,192 hits were obtained for putative peptide sequences that are similar to other proteins, whereas 28,201 sequences are unique. A total of 44,629 of transmembrane protein sequences were identified, of which 1729 belong to unique sequences.

Regarding the results obtained in the KEGG platform, 49,241 codes were assigned to metabolic routes in this database, of which 11,468 belong to a unique route.

### 2.4. EGGNOG Function Classification and Ontological Genes

**Figure 2:**
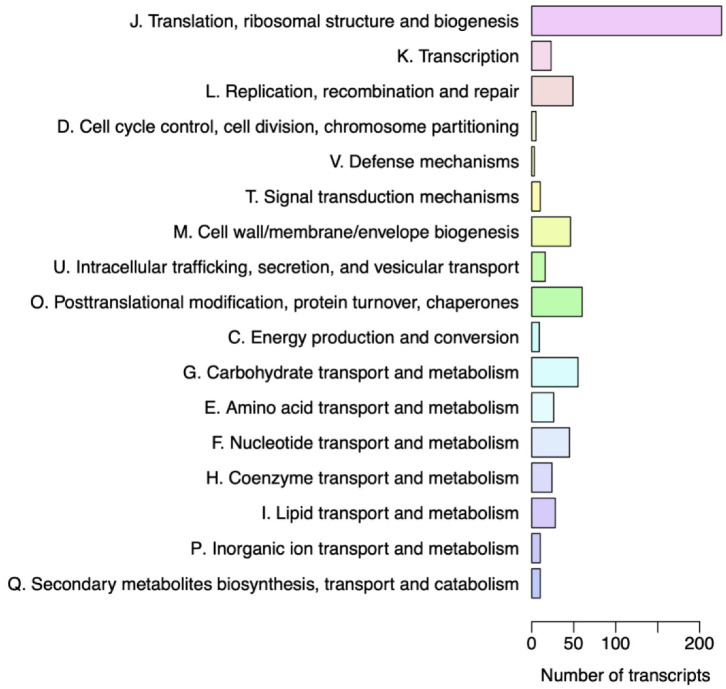
Functional classification of orthologous proteins obtained from the *M. bombycina* transcriptome. The figure shows that the highest number of hits was for translation, ribosomal structure, and biogenesis, whereas the lowest number of hits was obtained for the defense mechanism.

The description and functional classification of orthologous proteins was carried out in the EGGNOG database. Nine hundred and eighty-eight orthologous protein sequences were assigned from other species already annotated, such as that of *A. thaliana*. These sequences were classified into 17 different functional groups, among which the category translation, ribosomal structure, and biogenesis identified with section J. stand out, with 85% of the total hits (Figure 2). In contrast, the defense mechanisms category (V) contains the lowest number of sequences expressed with 5%.

**Figure 3:**
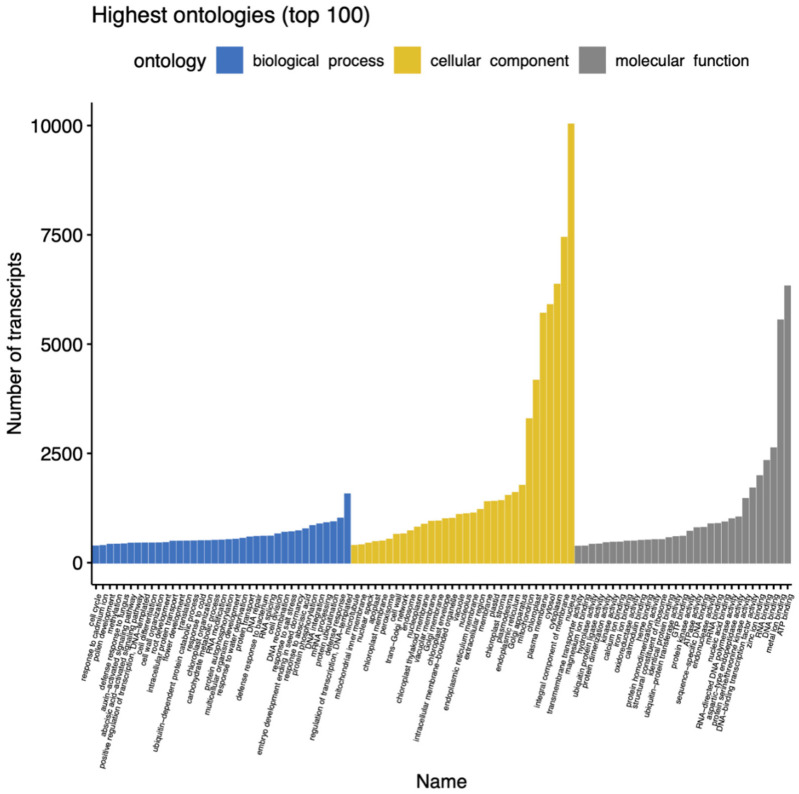
Classification of GO terms obtained from the *M. bombycina* transcriptome. In the biological process classification, the lowest number of hits was found in the cell cycle category, whereas more hits were found for the defense response. In the cell component classification, the lowest was found in the category of microtubule-related transcripts, and in a higher proportion were transcripts associated with nuclear location. Finally, in the classification of molecular function are transcripts related to membrane transport activity, and at a higher proportion are those transcripts related to ATP binding.

Regarding the classification of gene ontology (GO), a total of 43,640 transcripts were assigned, distributed in 99 different functional groups, and divided into three main categories: (a) cellular component, having around 60% of the total expressed gene sequences, of which, the highest number of hits correspond to genes related to nuclear function; (b) molecular function, corresponding to 30% of the transcripts, most of which are related to ATP binding, and (c) biological processes, which account for approximately 10% of the total hits; among them, the category with the highest number of Gene sequences are those related to the function of the regulation of transcription (Figure 3). GO terms were inferred using the annotation of genes that had blast matches with proteins belonging to the UniprotKB database.

For the distribution of hits in the Nr database (Figure S1), it was found that 90% of the functionally annotated transcripts coincide with *A. thaliana.* The second taxon most closely related to the annotations obtained was *Oryza sativa*, followed by other species that were in the repository. The remaining hits for which a match or a specific hit was not found may be the result of contaminations or genes that have not been well characterized.

### 2.5. Identification and Analysis of GLYI, GLYII, and GLYIII Genes

All members of the GLYI, GLYII, and GLYIII family of the *M. bombycina* transcriptome were analyzed in detail. Eight sequences similar to the GLYI protein family were identified; however, the putative protein sequence showed that only eight of these contain the Glyoxalase/Bleomycin-resistant domain (PF00903). These sequences were named and classified as MbGLYI-1 to MbGLYI-8 (Table 3). The size of the CDS for the GLYI was varied and with a length ranging from 564 bp (MbGLYI-3) to 1092 bp (MbGLYI-4), with an average size of 858 bp. In the same way, the size of the largest protein was 363 aa for MbGLYI-3, weighing 40.21 kDa, and the shortest was for MbGLYI-2, with 187 aa and 20.88 kDa. Most of these proteins (MgGLYI-2 to MgGLYI-8) have an acidic isoelectric point (pI), and only MgGLYI-1 is basic at pI 8.8 (Table 3). Subcellular location was also analyzed, found in chloroplasts, mitochondria, cytoplasm, and nucleus for MbGLYI-1. The putative protein sequence of MbGLYI-1 was aligned with the homologs of *A. thaliana* and *O. sativa* because these have been reported with nuclear activity and localization, having a protective activity for DNA when MG is present in the cell (Figure S2).

For the GLY II, four partial coding genes were obtained because these were missing parts towards the 5 ′ end. The putative partial sequences of aa contain the glyoxalase domains. The GLYII has an average mass of 35.91 kDa; MbGLYII-1 and MbGLYII-2 have a basic pI. The putative MbGLYII-3 protein has a slightly neutral pI, whereas the MbGLYII-4 pI is acidic. Its subcellular location is cytoplasmic, chloroplast, mitochondrial, and MbGLYII-3 is possibly nuclear because it presents a signal sequence in the cNLSMapper program [23] (Table 3). Regarding GLYIII (MbDJ-1), only one coding gene was found with a CDS of 1176 bp and a sequence of 392 aa, an acid pl of 5.5, and it is a cytoplasmic protein (Table 3).

### 2.6. Multiple Alignment and Domain Architecture

All putative proteins for glyoxalases from the *M. bombycina* transcriptome were analyzed to identify conserved domains. Figure S3 shows the alignment of all putative proteins and of other species already characterized; similarly, they all present the conserved domains and motifs and their catalytic sites. The putative proteins MbGLYI-1 and MbGLYI-2 present the domain of glyoxalase/bleomycin resistance protein/dioxygenase superfamily (PF00903), which is present in the dependent Zn^2+^; in the shaded boxes are the active aa and, in the star, the active site. For MbGLYI-3 to MbGLYI-8, the same domain (blue arrow) is present twice, making them Ni^2+^ dependent (Figure S3a); in the shaded boxes are the active aa and, in the star, the active site.

In the case of GLYII, although they are partial sequences, it was found that they all present the two domains of GLYII: (1) Metallo-beta-lactamase (PF00753) towards the terminal carboxyl (pink arrow) and (2) Hydroxyacylglutathione hydrolase C -terminal (PF16123) towards the amino terminal (green arrow); in addition, they contain the metal-binding sites (diamonds) and the conserved region THXHXDH/H/D/H, an active site (C/GHT), and seven conserved GSH-binding sites: (C/K(R)/F(Y)/Y/N/R/K) (Figure S3b).

In the case of GLYIII, only one sequence was found. The alignment with AtYL5S and AtDJ-1D shows that MbGLYIII-1 contains twice the DJ-1 domain (yellow arrows) that is characteristic of GLYIII and also presents the motifs conserved within these proteins (Figure S3c).

### 2.7. Hypothetical Modeling of the MbGLY Family

**Figure 4:**
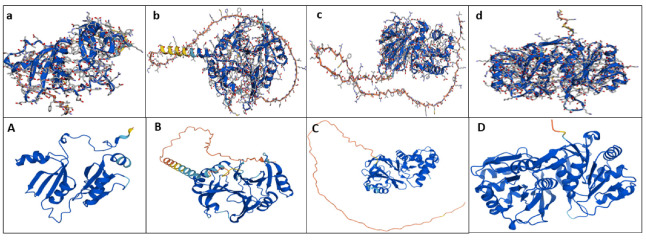
Comparison of the hypothetical modeling of the 3D structures of the putative proteins of MbGLYI, MbGLYII and MbGLYIII of *M. bombycina* with the proteins Glyoxalase I, Glyoxalase II and DJ1 of the AlphaFold platform. (**a**) MbGLYI-1 shows a Zn^2+^ dependent region. (**A**) Modeling of the Zn^2+^-dependent Lactoylglutathione lyase protein from *Brassica juncea* (O04885 LGUL_BRAJU). (**b**) Ni^2+^ dependent MbGLYI-3. (**B**) Lactoylglutathione lyase-dependent Ni^2+^ protein from *Zea Mays* (A0A1D6IYD6_MAIZE). (**c**) MbGLYII-1. (**C**) Modeling of the GLX2-4 protein from *Arabidopsis thaliana* (Q8LDW8, GLO2D_ARATH). (**d**) MbDJ-1. (**D**) *Arabidopsis thaliana* DJ1-D protein (Q9M8R4, DJ1D_ARATH).

To determine the folding of the composition of the aa of the MbGLY family, a homology model of the putative proteins of MbGLYI-1, MbGLYI-2, MbGLYII-1, and MbDJ-1 was constructed using the AlphaFold Colab program. Figure 4a shows the hypothetical structure of MbGLYI-1 with the glyoxalase/lactoylglutathione lyase domain for Ni^2+^-dependent MbGLYI-2 (Figure 4b). In the case of MbGLYII-1, the structure of the protein belongs to the family of metallo-β-lactamases (Figure 4c). In Figure 4d, the hypothetical modeling of MbDJ-1 is shown. We identified glyoxalases proteins from other plant species using the AlphaFold program and Uniprot Database (Figure 4A–D); (A) modeling of the Zn^2+^-dependent Lactoylglutathione lyase from *Brassica juncea*, (B) Lactoylglutathione lyase-dependent Ni^2+^ from *Zea Mays*, C) modeling of the GLX2-4 from *Arabidopsis thaliana*, and D) DJ1-D from *Arabidopsis thaliana*. In each model is shown in navy blue the highest degree of confidence (>90).

### 2.8. Phylogenetic Analysis of Glyoxalase from M. bombycina and Other Plants

**Figure 5:**
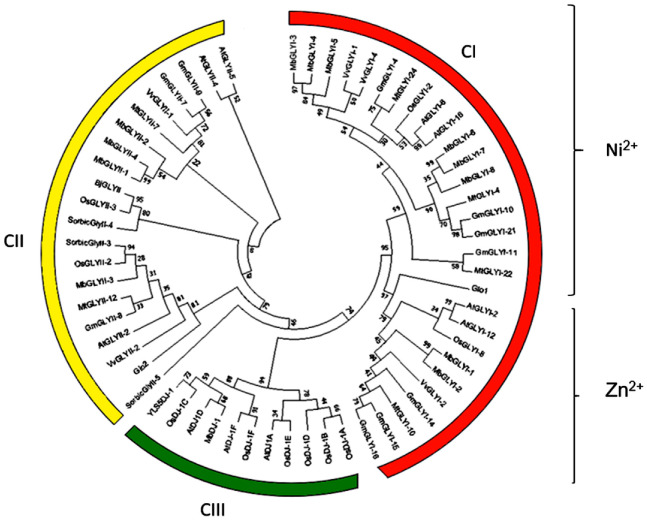
Phylogenetic analysis of the putative proteins MbGLYI, MbGLYII, and MbDJ-1. The phylogeny shows three groups; in CI (orange) are the putative protein sequences belonging to MbGLYI, which in turn are divided into Ni^2+^- and Zn^2+^-dependent. The CII group (yellow) contains the putative protein sequences of MbGLYII, whereas the putative protein sequence MbDJ-1 is located in group CIII (green).

Phylogenetic analysis for all members of MbGLY and other species shows three main groups (Figure 5). In the CI group (orange) are all the GLYI, and this group is divided into two subgroups, in the first one are those dependent on Zn^2+^ (MbGLYI-1 and MbGLYI-2), and they are closely related to OsGLYI-8, AtGLYI-2, AtGLYI-12, VvGLYI-2, and GmGLYI-16 (Figure 5). In subgroup 2 of the CI group, the Ni^2+^-dependent GLYI are found, which in turn are distributed in two groups. In the first group are MbGLYI-3, MbGLYI-4, and MbGLYI-5, very close to those of VvGlyI-1 and VvGlyI-4 *Vitis vinifera*. In contrast, MbGLYI-6, MbGLYI-7, and MbGLYI-8 are closely related to GmGLYI-10 and GmGLYI-21 of glycine *max*. In group CII (yellow), members of the MbGLYII family are seen. In subgroup 1 are MbGLYII-1, MbGLYII-2, and MbGLYII-4, which are closely related to AtGLYII-4 and VvGLYII-1, whereas in subgroup 2, MbGLYII-3 is related to VvGLYII-2. In group CIII (green) are the GLYIII, and here is located MbDJ-1, which has a close relationship with AtDJ1D of *A. thaliana.*

### 2.9. In Silico Analysis of Co-Expression and Expression Analysis (qRTPCR) of MbGLYI, MbGLYII, and MbDJ-1 Genes

**Figure 6:**
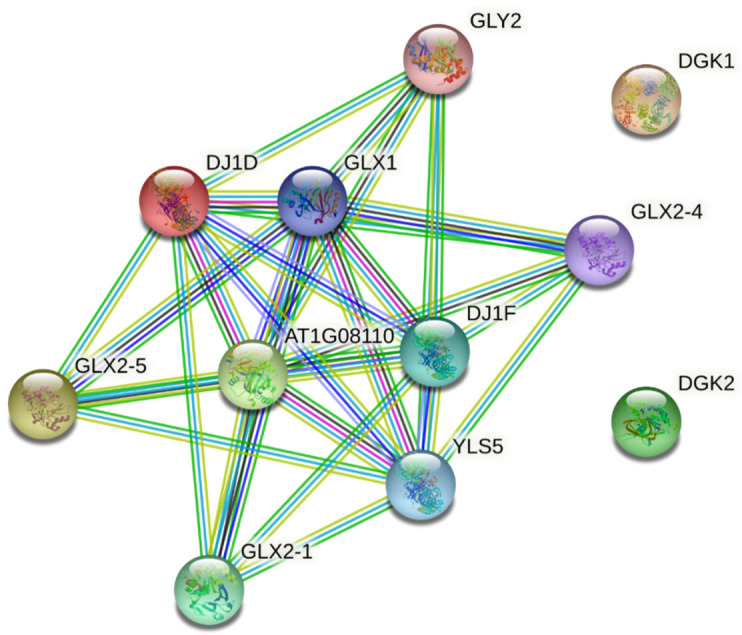
Map of protein–protein interactions for MbGLYI-1, MbGLYI-2, Glx2-5 MbGLYII-1, MbGLYII-4, and MbDJ1. The IPP network shows that all proteins interact with each other.

The IPP results were obtained from the orthologs for At1g8110 (MbGLYI-1), At1g67280 (MbGLYI-2), Glx2-5 (MbGLYII-1), Gly2 (MbGLYII-4), and DJ1D (MbDJ-1) (Figure 6). The five query proteins of M. bombycina interact with five others: 4 glyoxalases and a glutamine amido-transferase-like enzyme (YLS5). The analysis of the IPP in the STRING database shows that there are 11 nodes connected with 30 different edges, and the average node degree was 5.45, which means that at least one node has 5.45 interacting nodes. The IPP network shows that all proteins interact with each other (except for DGK1 and DGK2 which are the negative controls). The closest interaction between each protein is based on its score; thus, At1g08110 (MbGLYI-1) has an interaction with GLY2 with a score of 0.993. At1g67280 (MbGLYI-2) interacts with GLY2-I, GLY2-4, and GLY2-5, all with a score of 0.992. GLY2-5 interacts with At1g8110 (MbGLYI-1) with a score of 0.992. GLY2 interacts with At1g8110 (MbGLYI-1) with a score of 0.993. GLY-2 (MbGLYII-4) and DJI-D (MbDJ-1) interact with At1g8110 (MbGLYI-1) with a score of 0.965. Two sequences of the M. bombycina transcriptome encoding a two isoforms of Diacylglycerol kinase 2 was used as a negative control (DGK1 and DGK2).

**Figure 7:**
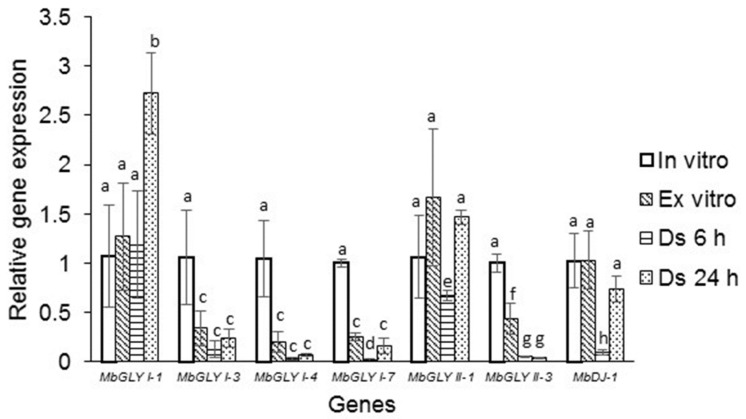
Expression analysis of MbGLYI, MbGLYII, and MbDJ-1 genes. The MbGLYI-1 gene shows a higher expression in the 24 h dehydration treatment, whereas MbGLYI-3, MbGLYI-4, and MbGLYI-7 expression decreased in all treatments compared to the control. For MbGLYII-1, a higher expression was presented in the ex vitro treatments and dehydration 24 h compared to the control. The expression in MbGLYII-3 is significantly lower in all treatments with respect to the control. In the case of MbDJ-1, the expression was maintained for the ex vitro treatments and dehydration at 24 h compared to the control; however, in the dehydration treatment 6 h, there was a significant decrease in the expression compared to the control. Different letters above the bars (**a**–**h**) represent a significant difference (*p* = 0.05) in Tukey test.

To analyze real-time expression, the plants generated in vitro were used as a control. The expression of MbGLYI-1 was 1.8-fold higher due to dehydration at 24 h, and in the other treatments, there were no significant changes compared to the control (Figure 7). For MbGLYI-3, 4, and 6, the expression levels decreased drastically compared to the control. With regard to MbGLYII-1, a slight increase in expression was observed in plants ex vitro and by dehydration at 24 h. For MbDJ-1, it was observed that the expression decreased by drying at 6 h compared to the control, and in the other conditions, there were no statistically significant changes in expression (Figure 7).

## 3. Discussion

*Mammillaria bombycina* belongs to the Cactaceae family. It is subject to special protection because it has been looted from its natural habitat to be used as an ornamental plant. However, the optimal conditions for its cultivation in vitro have been established, which has facilitated the study of some genes related to abiotic stress response [6]. The use of this species under in vitro conditions for its study at the gene level is of substantial relevance for obtaining genes of interest, as well as the knowledge of their regulatory and functional pathways.

In this work, the annotations of the de novo transcriptome of *M. bombycina* were made under in vitro conditions because there was no previous analysis of new-generation sequencing in this species. Therefore, *M. bombycina* is proposed as a model plant for study in cacti.

A total of 24 million clean reads were obtained, of which 84,975 transcripts found in public databases were noted, as shown in Table 2. Fifty-five percent are unique (47,406 unigenes), and most of these come from *A. thaliana* (Figure 4). Similar results were obtained from *Dimocarpus longan* [24] where, out of 13 samples, the authors generated 64,876,258 clean readings; however, they only obtained 68,925 unigenes. Similarly, in *Agave sisalana* [16], 276.8 million readings were obtained from 9 samples, recording 93,141 genes, of which 67,328 are unigenes. Therefore, our *M. bombycina* de novo transcriptome annotation is a robust and good-quality analysis.

Various conditions such as salinity, drought, high temperature, and exposure to heavy metals are responsible for producing abiotic stress and causing considerable losses in plant productivity [25]. Under stress conditions, plants trigger defense mechanisms based on the synthesis of different enzymes, among which glyoxalases stand out. Glyoxalases are responsible for biotransforming MG, a by-product of metabolism, which under normal conditions is produced in small amounts, whereas under some types of stress, its concentration increases up to six times, causing toxicity to the cell [13,26]. Thus, genetic studies of glyoxalases have been carried out in plants such as *Arabidopsis* [27], rice [28], sorghum [11], and soybeans [14]. However, until now, there have been no reports of *Gly* genes in cacti, despite their importance in adaptation to extreme climates. Hence, in this work, a search for GLY genes in the *M. bombycina* transcriptome was carried out.

**Figure 8:**
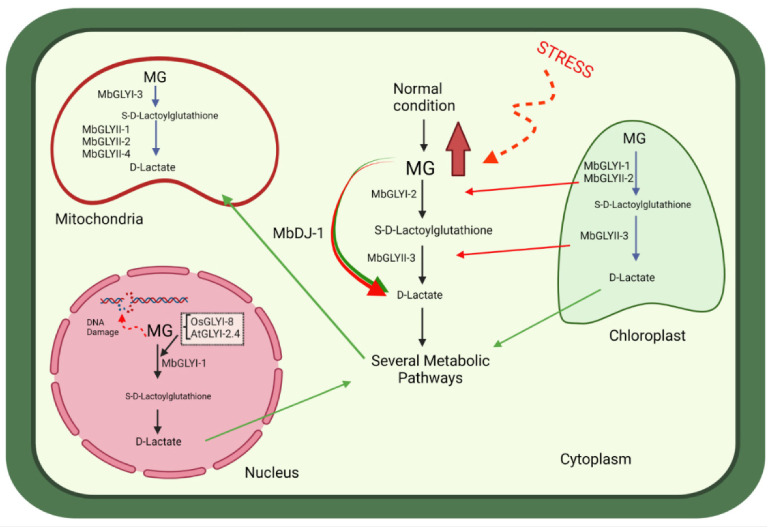
Hypothetical model proposed for the detoxification of MG through the glyoxalase pathway in various subcellular organelles in *M. bombycina*. The role hypothetically played by each of the putative proteins MbGLYI, MbGLYII, and MbDJ-1 in vegetal cells is observed.

In this study, 13 glyoxalase sequences were found: eight from GLYI (MbGLYI), four partial sequences from GLYII (MbGLYII), and one from GLYIII or DJ-1 (MbDJ-1). Bioinformatic analysis and cluster analysis showed that, for MbGLYI, there are two Zn^2+^-dependent genes and six Ni^2+^-dependent genes. *O. sativa*, *S. bicolor* and *A. thaliana* plants have also been reported to contain more Ni^2+^-dependent *GLYI* genes [11]. Lee et al. [29] argues that the higher number of Ni^2+^-dependent genes in plants is due to two evolutionary phenomena—gene duplication and divergence. The partial sequences of MbGLYII contain all the characteristics of glyoxalases II, and the putative protein sequence of MbDJ-1 showed all the characteristics of being a protein with functional activity, as described by Quigley et al. [30]. The cluster analysis showed that the analyzed sequences are closely related to glyoxalases that are expressed under different types of stress [11,14,28]. The putative protein sequence of MbGLYI-1 was found to contain a nuclear signal, indicating that it has the same function as the *A. thaliana* (AtGLYI-2) and *O. sativa* (OsGLYI-8) homologs because they have been reported with nuclear activity and localization [28]. With these results, a hypothetical model of the action of *M. bombycina* glyoxalases was proposed (Figure 8).

The hypothetical modeling of the 3D structures of the putative proteins of MbGLYI, MbGLYII and MbGLYIII of M. bombycine show a very similar structure with other glyoxalases models existing in the AlphaFold database (Figure 4). The confidence score in AlphaFold is produce by a per-residue confidence score (pLDDT) with values between 0 and 100. These values are showed in the modelling with different color; navy blue > 90, yellow < 70 and orange < 50 (this region may be unstructured in isolation). AlphaFold protein structure database provides a computational method to predict protein structures with atomic accury. This method has the advantage of predicting structures when a model structure is not known, it also shows great accuracy with models reported with other laboratory methods [31,32].

The IPP map shows that all MbGLY interact with each other and with other glyoxalases of the *Arabidopsis* genome and that all the proteins involved are related to abiotic stress resistance, including YLS5 [33]. The expression analysis shows that the MbGLY genes have a similar pattern of expression to those reported in other plant species [11,14,34]. However, it has also been observed that the expression of *GLYs* genes responds differently depending on the plant and type of stress [27]. With these results, a line of research is opened regarding the behavior of this gene family in *M. bombycina* under in vitro, ex vitro, and dehydration conditions.

## 4. Materials and Methods

### 4.1. Vegetal Material

*M. bombycina* seedlings were obtained from the in vitro Germplasm Bank of the Plant Biotechnology Unit of the Universidad Autónoma de Aguascalientes (Aguascalientes, México). The vegetal material was propagated on MS culture medium [35] with 0.5 m/L of BA (Benzyladenine) [6].

### 4.2. RNA Extraction and Sequencing by the Illumina Method

Total RNA was extracted from the aerial parts of *M. bombycina*. The tissue was immediately cut and pulverized with liquid nitrogen. Two g of powdered tissue were used, and RNA was extracted using the commercial Trizol kit (Invitrogen) following the manufacturer’s recommendations. RNA purity was verified using an Agilent Bioanalyzer electropherogram (Agilent Technologies, Santa Clara, CA, USA). The construction of the libraries was done with an Illumina TruSeq Stranded mRNA Sample Preparation Kit. Sequencing was carried out with NextSeq 500 equipment from the Illumina company, using a NextSeq 500/550 v2.5 High Output Kit, at the Biotechnology Institute of UNAM at the Unidad Universitaria de Secuenciación Masiva de Datos y Bioinformática (UUSMB) (Cuernavaca, Morelos, Mexico).

### 4.3. De Novo Transcriptome Assembly and M. bombycina Functional Annotation

The FastQC tool (http://www.bioinformatics.babraham.ac.uk/projects/fastqc (accessed on 14 April 2021)) was used to evaluate the quality of raw data from high-throughput sequencing, whereas trimming and filtering of the readings were performed using the Fastp program (http://opengene.org/fastp/fastp (accessed on 15 April 2021)). Digital normalization and de novo transcriptome assembly were carried out using Trinity 2.0.5 software with default values [36]. The annotation of the putative genes assembled with the Trinity program was performed using a BlastT analysis in several databases, including NCBI (not redundant; Nr) (ftp://ftp.ncbi.nih.gov/blast/db/ (accessed on 17 April 2021)), the Swissprot-Uniprot database [37], and the Kyoto encyclopedia of genes and genomes (KEGG) [38], with an E cut-off value set at 10-5. Analysis of the open reading frames (ORFs) of genes was done with the program Transdecoder v.2.0.1 (https://github.com/TransDecoder (accessed on 17 April 2021)), with a cut-off value E of 10-5 (http://transdecoder.sourceforge.net/ (accessed on 17 April 2021)). The remaining functional annotation was carried out with Trinotate (https://trinotate.github.io/ (accessed on 18 April 2021)), through various programs such as Hmmer v.3.1b1 [39], Tmhmm v.2 [40], SignalP v.4.1 [41], GOseq [42], and EGGNOG v.3.0. [43]. The summary of results was made from the trinotate.xls database from R version 3.5.0 (https://cran.r-project.org/bin/windows/base/old/3.5.0/ (accessed on 21 April 2021)).

### 4.4. In Silico Analysis of Glyoxalase Genes (GLY)

The identification of the putative gene sequences of GLYI, GLYII, and GLYIII was obtained from the *M. bombycina* transcriptome annotations and confirmed by Blastx in the NCBI database (https://www.ncbi.nlm.nih.gov/ (accessed on 22 April 2021)). The identification of the domains was carried out in the Pfam database (http://pfam.xfam.org/ (accessed on 22 April 2021)) and Prosite (https://prosite.expasy.org (accessed on 22 April 2021)).

The physicochemical analysis of the putative amino acid (aa) sequences was performed with the ProtParam tool [https://web.expasy.org/protparam (accessed on 4 May 2021)] [44]. The subcellular localization was predicted with the WolfPSORT programs [https://www.genscript.com/wolf-psort.html (accessed on 4 May 2021)] [45], CELLO [http://cello.life.nctu.edu.tw/ (accessed on 4 May 2021)] [46], and LOCALIZER [http://localizer.csiro.au/ (accessed on 4 May 2021)] [47]. The signal sequence was predicted using the cNLSMapper program [http://nls-mapper.iab.keio.ac.jp/cgi-bin/NLS_Mapper_form.cgi (accessed on 5 May 2021)] [23].

Multiple sequence alignment was carried out using ClustalW [48] and visualized with Jalview [49].

The construction of phylogenetic trees without roots was carried out using the MEGA version 7 program with the UPGMA method and 1000 bootstrap replicas [50]. GLY sequences from other organisms containing the N-terminal lactoylglutathione lyase domain were analyzed, such as *Vitis vinifera* (VvGLY), *Oryza sativa* (OsGLY), *Arabidopsis thaliana* (AtGLY), *Glicine max* (GmGLY), *Medicago truncatula* (MtGLY), *Brassica juncea* (BjGLY), and human (GloI). The same procedure was performed for GLYII proteins with the metallo-β-lactamase domains and the C-terminal Hydroxyacylglutathione hydrolase domain; *Vitis vinifera* (VvGLYII), *Oryza sativa* (OsGLYII), *Arabidopsis thaliana* (AtGLYII), *Glicine max* (GmGLYII), *Medicago truncatula* (MtGLYII), *Brassica juncea* (BjGLYII), and *Sorgum bicolor* (SorBiGLYII). For DJ-1 proteins, construction was carried out with GlyIII proteins with the DJ-1 / PfpI domain; *Homo sapiens* (HsDJ-1), *Oryza sativa* (OsDJ-1), *Arabidopsis thaliana* (AtDJ-1 and YLS5DJ-1).

The prediction of the 3D structure modeling of the putative protein sequences was performed with the program AlphaFold Colab [https://colab.research.google.com/github/deepmind/alphafold/blob/main/notebooks/AlphaFold.ipynb (accessed on 4 January 2022)] (Jumper et al. 2021, Varadi et al. 2021). The protein–protein interaction analysis was carried out using the STRING platform (https://string-db.org/ (accessed on 26 December 2021)) [51].

### 4.5. Expression Analysis

From the coding sequences for GLYI, GLYII, and GLYIII in the transcriptome, oligonucleotides were designed (Supplementary Material Table S1). The 25S ribosomal subunit was used as a reference gene with the primers [52].

Seedlings of *M. bombycina* 4 cm tall and one year old were used, testing the following treatments in triplicate: (1) in vitro seedlings (control), (2) ex vitro seedlings kept in a greenhouse, (3) in vitro seedlings dehydrated for 6 h, and (4) in vitro seedlings dehydrated for 24 h. For each treatment, nine seedlings were used in each one of them. Total RNA was extracted using the TRIzol kit (Invitrogen) following the manufacturer’s recommendations. The cDNA synthesis was carried out with the Radiant 1-step kit.

Real-time expression analysis was performed using the SYBER Green kit (Applied Biosystem, Carlsbad, CA, USA). The reaction mixture was 5 µL of SYBER Green reagent, 0.3 µM of each oligonucleotide, 100 ng of cDNA, and 3 µL of sterile distilled water for a final reaction of 10 µL. Statistical analysis was carried out using the 2^−∆∆C^^T^. method, followed by a one-way ANOVA (*p* < 0.05) and a Tukey test in the Statistic 10.0 program [53].

### 4.6. Sequence Data

These sequence data have been submitted to the SRA/GenBank databases under accession number: PRJNA764261.

## Figures and Tables

**Table 1:** Statistical analysis of *M. bombycina* transcriptome assembly evaluation.

Counts of Transcripts, etc.	
Total trinity ‘genes’	47,406
Total trinity transcripts	79,881
Percent GC	44.22
Stats based on ALL transcript contigs	
Contig N10	3539
Contig N20	2742
Contig N30	2227
Contig N40	1870
Contig N50	1574
Median contig length	641
Average contig	981.61
Total assembled bases	78,412,335
Stats based on ONLY LONGEST ISOFORM per ‘GENE’	
Contig N10	3337
Contig N20	2547
Contig N30	2077
Contig N40	1730
Contig N50	1417
Median contig length	418
Average contig	794.74
Total assembled bases	37,675,612

**Table 2:** Statistical analysis of *M. bombycina* transcriptome annotations.

	Unique	Total
gene_id	47,406	84,975
transcript_id	79,881	84,975
prot_id	44,629	44,629
prot_coords	32,539	44,629
TmHMM	1729	44,629
sprot_Top_BLASTX_hit	38,175	43,640
gene_ontology_BLASTX	9638	42,361
Kegg	11,468	39,241
sprot_Top_BLASTP_hit	28,201	34,192
gene_ontology_BLASTP	8947	33,218
Pfam	25,403	30,825
gene_ontology_Pfam	1547	19,238
SignalP	1284	2115
eggnog	205	988

**Table 3:** Physicochemical characterization and cellular localization of MbGLYI, MbGLYII, and MbDJ-1 putative genes found in the *M. bombycina* transcriptome.

Name Gene	Transcript	Transcript Length (pb)	CDS (pb)	Protein			Localization
				Putative Protein	MW (kDa)	Pl	
MbGLYI-1	TRINITY_DN14362_c0_g1_i4	1161	708	235	26.35703	8.82	Chlo ^ac^, nucl ^b^
MbGLYI-2	TRINITY_DN14362_c0_g1_i7	1023	564	187	20.88262	5.43	Chlo
MbGLYI-3	TRINITY_DN14567_c0_g1_i2	1701	1092	363	40.21782	6.64	Chlo ^ac^, mit ^b^
MbGLYI-4	TRINITY_DN14567_c0_g1_i5	2457	792	263	29.53397	5.13	Nucl ^a^, cyt ^b^
MbGLYI-5	TRINITY_DN14567_c0_g2_i1	1400	1071	356	39.31778	5.80	Chlo ^abc^
MbGLYI-6	TRINITY_DN15304_c0_g1_i1	1219	870	289	32.68124	5.32	Nucl ^a^ Nucl ^c^
MbGLYI-7	TRINITY_DN15304_c0_g1_i2	1024	903	300	33.65052	5.70	Cyto ^ac^
MbGLYI-8	TRINITY_DN15304_c0_g2_i1	1386	870	289	32.43995	5.26	Cyto ^ac^
MbGLYII_1	TRINITY_DN9859_c0_g1_i1	1112	NA	334	37.19777	8.72	Chlo ^a^,mito ^c^
MbGLYII_2	TRINITY_DN9859_c0_g1_i2	1073	NA	321	35.72691	8.45	Chlo ^ab^, mito ^c^
MbGLYII_3	TRINITY_DN14689_c0_g1_i3	1306	NA	348	38.42322	7.30	Chlo ^a^, cyto ^b^ Nucl ^c^
MbGLYII_4	TRINITY_DN15347_c0_g1_i3	1079	NA	290	32.34300	6.55	Cyto ^a^, mit ^c^
MbDJ-1	TRINITY_DN10140_c0_g1_i1	1617	1176	392	41,974.14	5.57	Cyto ^ac^

Abbreviations: CDS coding DNA sequence, kDa kilo Daltons, pI isoelectric point, Chlo chloroplast, Cyto cytoplasm, NA does not apply. Prediction of the location: ^a^: Wolf Psort, ^b^: Localizer and ^c^: CELLO. pIs and molecular weights were obtained by using the ProtParam program.

## Data Availability

Not applicable.

## Supplementary Materials

The following are available online at https://www.mdpi.com/article/10.3390/plants11030399/s1, Table S1: Primers used in this study, Table S2: Information obtained from the sequencing performed on the Next Seq 500 equipment, in which a 2X75 cycle configuration was used, Table S3: Comparative table of the results obtained from the sequencer and those obtained after trimming and filtering sequences with the program, FastP, Table S4: Table of the percentages retained from clipping sequences with the FastP program, Figure S1: Distribution of hits in the *M. bombycina* transcriptome Nr. database, Figure S2: Alignment of MbGLYI-1, AtGLYI-2, and OsGLYI-8, Figure S3: Multiple alignment and architecture of the MbGLYI, MbGLYII, and MbDJ-1 domains.
