# Supplementary material for: De Novo Transcriptome of Mammillaria bombycina (Cactaceae) under In Vitro Conditions and Identification of Glyoxalase Genes
Source: Plants (Basel). 2022 Jan 31;11(3):399. doi: 10.3390/plants11030399 (PMC8838482; doi:10.3390/plants11030399)
Supplement: Supplementary file 1 [file plants-11-00399-s001.zip › plants-1483185-supplementary.pdf]

SUPPLEMENTARY MATERIAL

Table S1. Primers used in this study

GEN	FORWARD	REVERSE
**MBGLYI-1**	CTATTTCTTCGTTTAGGTGC	GAGAACAACTGAAACCGAT
**MBGLYI-3**	TCTAATAAAGGCCAAAGGGG	CACAATGGCTCAGGTGTAGG
**MBGLYI-4**	ATCTAATAAAGGCCAAAGGG	TAACTTGGCACAATGGCTCA
**MBGLYI-7**	AATGGTTGAAGACATTCGTG	ATTACTTGGCAGAGTGGTTC
**MBGLYII-1**	CGTCCTTAACAGATGAGACA	TACTGCGTAAATGAGCTACG
**MBGLYII-3**	AGCCGACTAGAGTTTATTGT	CAATGGTTGAAGGAACAGTT
**MBDJ-1**	GGAGCCGGAGCCAATAGACC	TCACTCAGAATGTTACACGGATGC

Sequencing with the Illumina HiSeq 500 Platform

Table S2. Information obtained from the sequencing performed on the Next Seq 500 equipment, in which a 2X75 cycle configuration was used (MB1R1/R2, MB2R1/R2, MB3R1/R2 belong to the Forward and Reverse section respectively).

SAMPLE	SIZE GB	READINGS OBTAINED	LENGTH OF READINGS	%GC
**MB1R1**	1.4	6,089,759	76	49
**MB1R2**	1.25	6,089,759	76	50
**MB2R1**	1.92	9,289,382	76	51
**MB2R2**	1.92	9,289,382	76	53
**MB3R1**	1.79	8,667,632	76	52
**MB3R2**	1.79	8,667,632	76	53

FastQC analysis, Trimming and filtering of the sequences obtained

Table S3. Comparative table of the results obtained from the sequencer and those obtained after trimming and filtering sequences with the program, FastP.

	Readings Obtained			FastP		
	**Readings**	**Length**	**%GC**	**Readings**	**Length**	**%GC**
**MB1R1**	6,089,759	76	49	6015549	15-76	49
**MB1R2**	6,089,759	76	50	5583001	15-76	49
**MB2R1**	9,289,382	76	51	8885020	15-76	50
**MB2R2**	9,289,382	76	53	7924826	15-76	50
**MB3R1**	8,667,632	76	52	8019521	15-76	49
**MB3R2**	8,667,632	76	53	7373640	15-76	49

Table S4. Table of the percentages retained from clipping sequences with the FastP program

	**Sequencer**	**% Obtained FastP**
MB1	6,089,759	98.7%
MB2	9,289,382	95.6%
MB3	8,667,632	92.52%

Figures

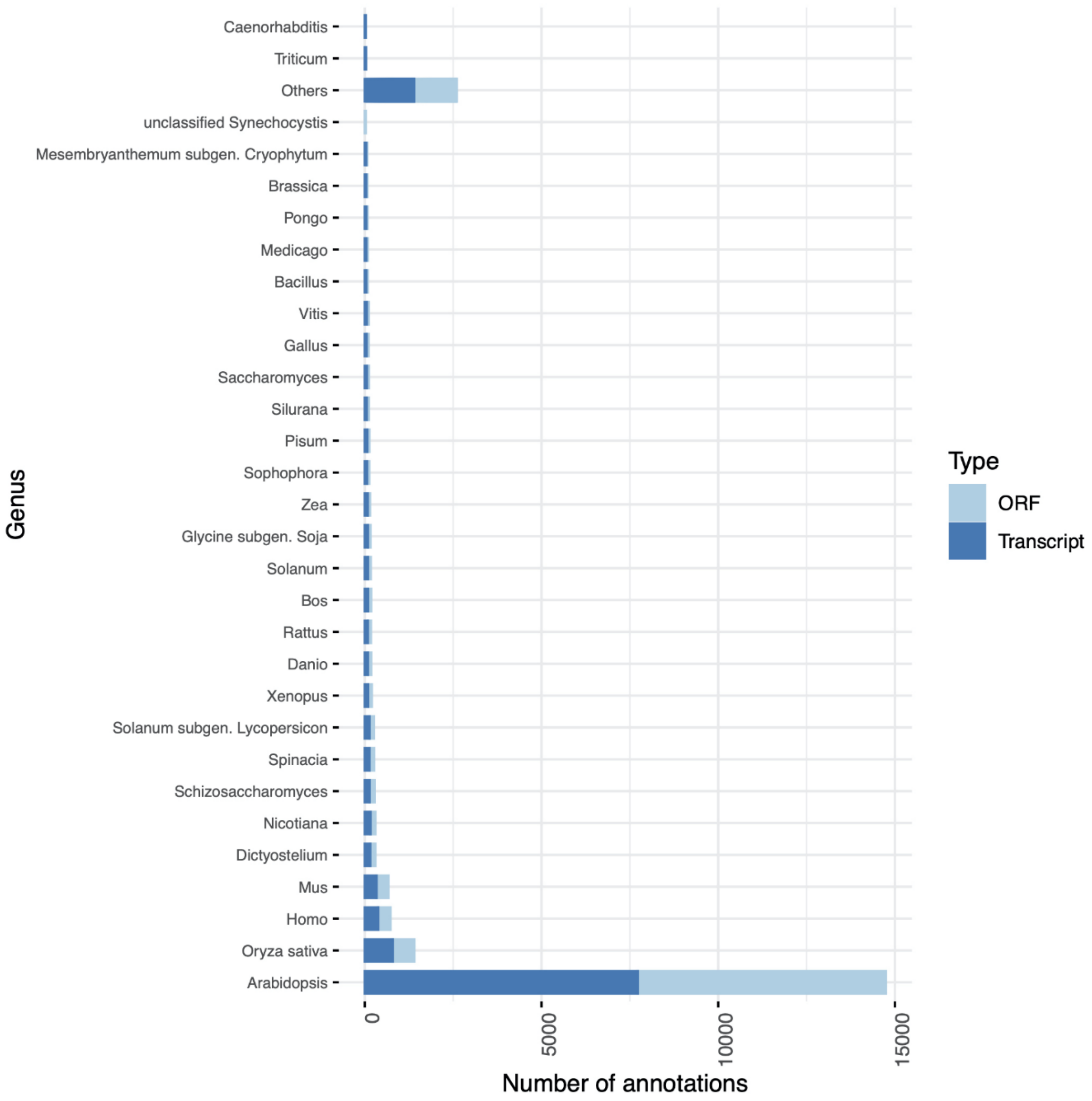

Figure S1 Distribution of hits in the *M. bombycina* transcriptome Nr database. Most of the hits obtained come from homologs of the species *A. thaliana.*

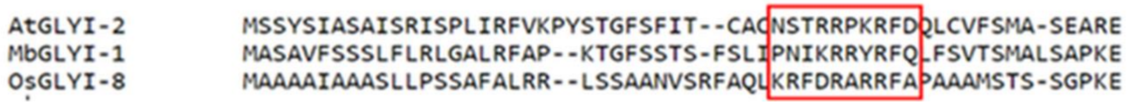

Figure S2. Alignment of MbGLYI-1, AtGLYI-2, and OsGLYI-8. The alignment shows that all three sequences have a nuclear signaling region (red box).

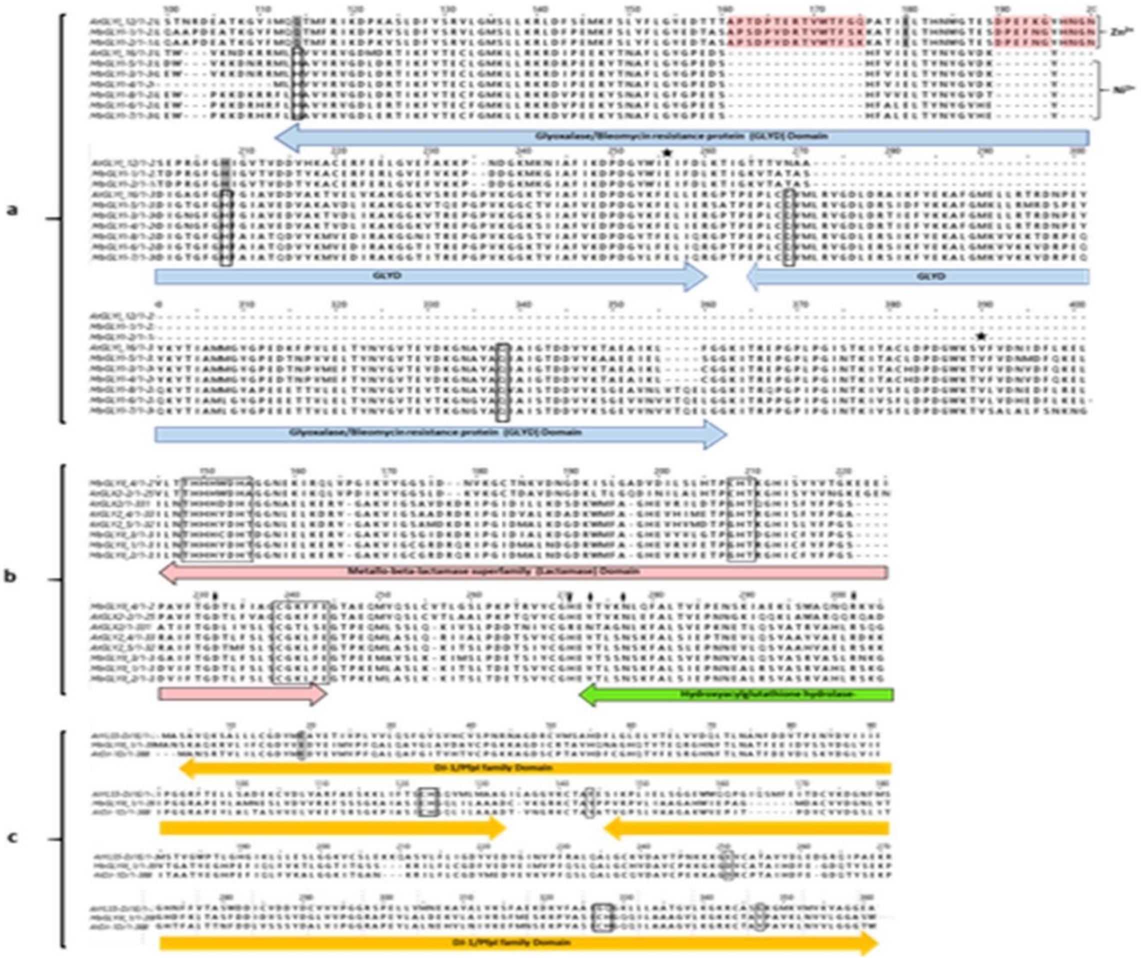

Figure S3. Multiple alignment and architecture of the MbGLYI, MbGLYII, and MbDJ-1 domains. a) Alignment of the MbGLYI dependent on Zn^{2+}; enclosed in the rectangle are the metal junction aa and with the star the catalytic site; on the right is the architecture of the domain. b) MbGLYII alignment; the arrows indicate the domains; enclosed in the rectangle are

the metal-binding aa and the catalytic site with the star; to the right is the architecture of the domain. c) MbDJ-1 alignment; enclosed in the rectangle are the catalytic sites found in each of the domains indicated by the arrows.
